# The Role of TRiC-enhanced Actin Folding in Leber Congenital Amaurosis

**DOI:** 10.18502/jovr.v18i1.12726

**Published:** 2023-02-21

**Authors:** Silke Berger, Peter D. Currie, Joachim Berger

**Affiliations:** ^1^Australian Regenerative Medicine Institute, Monash University, Clayton, Australia; ^2^Victoria Node, EMBL Australia, Clayton, Australia

**Keywords:** Actin, cct5, Leber Congenital Amaurosis, Retina, TRiC, Zebrafish

## Abstract

**Purpose:**

Mutations in TCP-1 ring complex (TRiC) have been associated with Leber Congenital Amaurosis (LCA). TRiC is involved in protein folding and has 8 essential subunits including CCT5. Herein, we studied the retina of TRiC mutant zebrafish to evaluate the possible role of impaired actin and tubulin folding in LCA.

**Methods:**

The *cct5 ${}^{tf212b}$* retina was histologically studied using Toluidine Blue staining as well as TUNEL, BrdU-labeling, and Phalloidin assays. Retinal organisation was assessed by quantification of the cellularity utilising DAPI.

**Results:**

Laminar organization of *cct5 ${}^{tf212b}$* retinas was intact. Enhanced apoptosis throughout the *cct5 ${}^{tf212b}$* retina was not compensated by higher proliferation rates, leaving the *cct5 ${}^{tf212b}$* retina smaller in size. Quantification of retinal layer cellularity demonstrated that specifically the numbers of the amacrine and the retinal ganglion cells were depleted, suggesting that the *cct5 ${}^{tf212b}$* retina was not uniformly affected by the reduced actin folding.

**Conclusion:**

Whereas the current literature suggests that LCA is predominantly affecting retinal photoreceptor cells and the retinal pigment epithelium, *cct5 ${}^{tf212b}$* analyses demonstrated the important role of folding of actin by TRiC, suggesting that *cct5 ${}^{tf212b}$* is a useful tool to specifically analyze the role of F-actin filaments in the context of LCA.

##  INTRODUCTION

Leber Congenital Amaurosis (LCA) is an early-onset congenital retinal dystrophy causing severe visual impairment. Over 38 disease-causing variants involving various pathological mechanisms have been associated with LCA, which consequently presents with a broad clinical spectrum, typically including poor to near-absent pupillary responses, nystagmus, photophobia, and severe visual impairment.^[1]^ Diagnosis is made within the first year of life usually based on extinguished electroretinograms, detecting little if any retinal activity.

Compound heterozygous missense mutations in *CCT2* have been identified in individuals suffering from LCA.^[2]^ Computational modeling combined with biochemical studies suggested that these mutations combined induce partial, and not complete, loss of TRiC functionality.^[2]^ *CCT2* is a subunit of the eukaryotic TCP-1 ring complex (TRiC, also called chaperonin containing TCP-1 [CCT]); an ATP-driven chaperonin that aids mis- or unfolded proteins in their folding. TRiC has a barrel-shaped structure with two back-to-back rings, each comprising of eight paralogous subunits (CCT1 - 8).^[3]^ Whereas several substrates have been described for TRiC,^[4]^ actin as well as all α - and β -tubulin are the main folding substrates.^[5,6]^ Accordingly, genetic analysis of TRiC in the zebrafish vertebrate model system *in vivo* revealed that loss of TRiC function leads to severe muscle and neuronal defects provoked by deficits in folding of actin and tubulin.^[7,8]^ The notion from structural data that loss of a single subunit leads to full loss of TRiC function was confirmed by genetic analysis of single and compound mutants.^[3,7]^ In a forward genetic screen, the zebrafish mutant *tf212b* was isolated based on its abnormal locomotion behavior and later reported to harbor a missense mutation leading to G422V replacement within the Cct5 subunit of TRiC.^[7,9]^ In contrast to mutants with a full loss of TRiC function, the missense mutation in *cct5 ${}^{tf212b}$* leads to actin misfolding by TRiC, whereas tubulin folding is not affected.^[7]^

In order to assess the role of impaired actin folding in retinal development, the retina of *cct5 ${}^{tf212b}$* was studied. As tubulin folding is intact in *cct5 ${}^{tf212b}$* homozygotes, defects of the retina within *cct5 ${}^{tf212b}$* homozygotes can be attributed to impaired actin folding. According to the impaired folding of actin within *cct5 ${}^{tf212b}$ *, retina analyses revealed that the formation of F-actin filaments is reduced in the *cct5 ${}^{tf212b}$* retina. Although proliferation was enhanced within the ciliary marginal zone, the retina in *cct5 ${}^{tf212b}$* mutants was smaller in size, likely due to enhanced apoptosis. Further cellularity quantification of the retinal layers revealed a reduction number of retinal ganglion cells and amacrine cells, indicating that these cell types are specifically affected by reduced actin folding.

##  METHODS

### Maintenance and Genotyping of Zebrafish

The zebrafish lines *cct5 ${}^{tf212b}$* and *cct4 ${}^{-14}$* were maintained in the TU (Tübingen) background under the ERM/22161 breeding license of the Monash Animal Service.Genotyping of *cct4 ${}^{-14}$* mutants was achieved by using the oligonucleotides cct4_F (5'-cccgagtttcttgaccacgttg) and cct4_R (5'-ctccacctcgctctgctctaag) in a PCR resulting in a 131 bp amplicon for the *cct4 ${}^{-14}$* allele. For *cct5 ${}^{tf212b}$ ,* the oligonucleotides Cct5_Bst_F (5'-acctggtgagggataatcgtatccagtat) and gCCT5_R1 (5'-cgggcactgaacacaagacaatc) were used in a PCR followed by restriction digestion with BstXI as reported^[7]^.

### Immunohistochemistry and Histological Analysis

Immunohistochemistry as well as the stains with Toluidine Blue, DAPI, and H&E were performed according to standard methods. Only retinal cross sections with optic nerve were analyzed. Retinal areas were measured on H&E or Toluidine Blue-stained cross sections using the software ImageJ. BrdU labeling was performed by soaking larvae in 1% bromodeoxyuridine (BrdU) dissolved in fish water followed by 14 µm cryostat cross sectioning and treatment with fluorescein conjugated BrdU monoclonal antibodies (BMC9318, Merck) as described.^[10]^ Apoptosis was detected using the *in situ* Cell Death Detection Kit as recommended by the manufacturer (Roche). All fluorescence images were recorded on a Zeiss ImagerZ1 fluorescence microscope (Zeiss, Germany). Images within figures show representatives of a minimum of six analyzed larvae per genotype.

### Comparison of F-actin Levels

F-actin was detected on 14 µm cryostat cross sections with phalloidin conjugated with AlexaFluor-568 (A12380, Life Technologies). To compare F-actin levels, phalloidin-stained sections were imaged on a Zeiss ImagerZ1 fluorescence microscope (Zeiss, Germany) under constant conditions and subsequently analyzed for their brightness values. Utilizing the software Fiji, the area of the inner plexiform layer (IPL) was selected and the mean of all grey values of the pixels within this area was measured, resulting in a single grey value per quantified IPL. To enable comparison of the brightness of the IPL from different genotypes, obtained grey values were rescaled to siblings set to 100%. To rescale grey values of siblings, measured grey values (A ${}_{1}$ to A ${}_{n}$ ) of individual IPL were multiplied by 100 and divided by the average of all measured grey values of siblings using $\frac{A_{i}\times 100}{\sum_{i=1}^{n}A_{i}/n}$ . To normalize values of mutants, measured grey values (B ${}_{1}$ to B ${}_{n}$ ) of individual IPL were multiplied by 100 and divided by the average of the measured grey values of the siblings using $\frac{B_{i}\times 100}{\sum_{i=1}^{n}A_{i}/n}$ . Only IPL from retinal cross sections were analyzed that showed the optic nerve. Ten IPL were analyzed per genotype (*n* = 10).

### Quantification of Cellularity of Retinal Layers

At six days post fertilization (dpf), transverse sections were stained with DAPI to label cell nuclei. Subsequent to imaging using a Zeiss ImagerZ1 fluorescence microscope (Zeiss, Germany), images were converted to 8-bit format and adjusted for contrast, brightness, and threshold. The outer nuclear layer, the inner nuclear layer, and the ganglion cell layer were distinguished based on their separation by the plexiform layers. The inner nuclear layer was further subdivided into a basal and an apical layer based on the difference in the fluorescence intensity of their nuclei. All DAPI-marked cell nuclei in individual layers were counted as described earlier.^[11]^ Five sections per genotype on the level of the optic nerve were included in the quantification (*n* = 5).

### Statistical Analysis

Significance between two groups was determined by Student's *t*-test. Statistical significance was calculated using the software Prism (GraphPad Software). Presented data are mean ± standard error of the mean (SEM).

**Figure 1:**
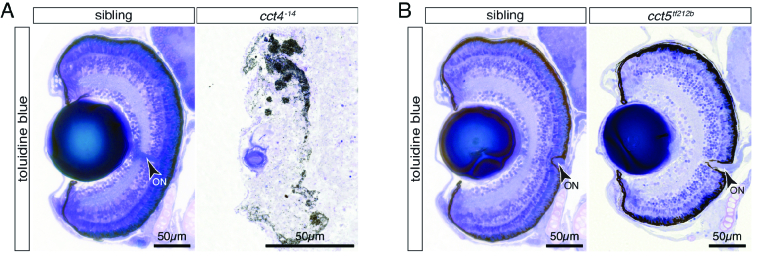
The eye develops in *cct5 ${}^{tf121b}$* but not *cct4 ${}^{-14}$* mutants. (A) Toluidine Blue-stained sections of six-dpf-old siblings revealed the typical layered structure of the retina. In contrast, the eye of TRiC loss-of-function *cct4 ${}^{-14}$* mutants was severely degraded and only rudimentary structures of lens and pigmented retina were recognisable. (B) At six dpf, homozygotes of the missense mutant *cct5 ${}^{tf212b}$* featured a structured eye and the layering of the retina appeared comparable to siblings. Arrowheads indicate optic nerves (ON).

**Figure 2:**
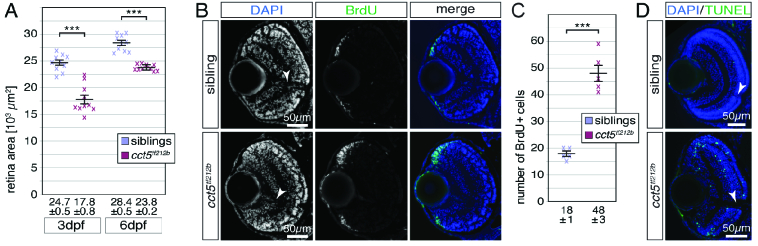
The retina of *cct5 ${}^{tf121b}$* mutants is smaller and characterised by enhanced proliferation and apoptosis. (A) Quantification of the retina sizes revealed that the retinal area of *cct5 ${}^{tf121b}$* homozygotes was significantly smaller compared to siblings. The mean retinal area of *cct5 ${}^{tf121b}$* homozygotes was 17.8 ± 0.8 ( × 10^3^ µm^2^) at three dpf and 23.8 ± 0.2 ( × 10^3^ µm^2^) at six dpf compared to siblings that featured mean retinal areas of 24.7 ± 0.5 ( × 10^3^ µm^2^) at three dpf and 28.4 ± 0.5 ( × 10^3^ µm^2^) at six dpf; *n* = 10 per genotype and stage. (B) In contrast to three-dpf-old siblings, more BrdU-positive cells (green) were detected at the periphery of the retina within the ciliary marginal zone of *cct5 ${}^{tf121b}$* homozygotes. (C) Quantification of the number of BrdU-positive cells within individual retinas revealed that significantly more cells were proliferating within the ciliary marginal zone of *cct5 ${}^{tf121b}$* homozygotes at three dpf. Whereas *cct5 ${}^{tf121b}$* had 48 ± 3 BrdU-positive cells per retinal cross section, 18 ± 1 were proliferating in siblings (*n* = 5). (D) In contrast to the retina of three-dpf-old siblings, in which apoptotic cells were rarely labelled by the TUNEL assay (green), apoptosis was frequently observed throughout the entire retina of *cct5 ${}^{tf121b}$* homozygotes. Data are mean ± SEM; ***P < 0.001 by Student's *t*-test. Arrowheads indicate optic nerves (ON).

**Figure 3:**
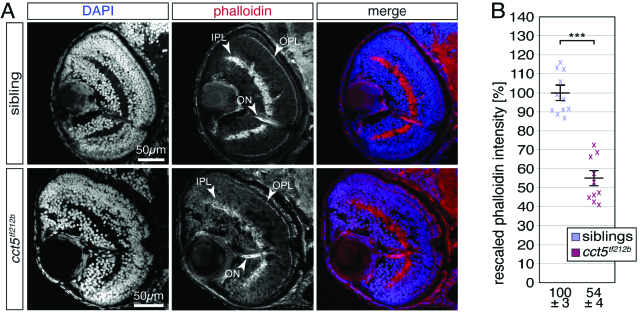
F-actin is significantly reduced in the retina of *cct5 ${}^{tf121b}$* mutants. (A) At three dpf, F-actin was labelled with phalloidin (red) on cross sections counterstained with DAPI (blue). Whereas the inner and outer plexiform layers of the retina (IPL and OPL, respectively) were prominently stained by phalloidin within siblings, the signal from *cct5 ${}^{tf121b}$* homozygotes appeared weaker. Phalloidin also marked the optic nerve (ON). (B) Quantification of the brightness of the phalloidin signal within the IPL revealed that *cct5 ${}^{tf121b}$* homozygotes showed a significant reduction in signal intensity to 54 ± 4% in relation to their siblings, which were rescaled to 100 ± 3%. Data are mean ± SEM; ***P < 0.001 by Student's *t*-test; *n* = 10.

**Figure 4:**
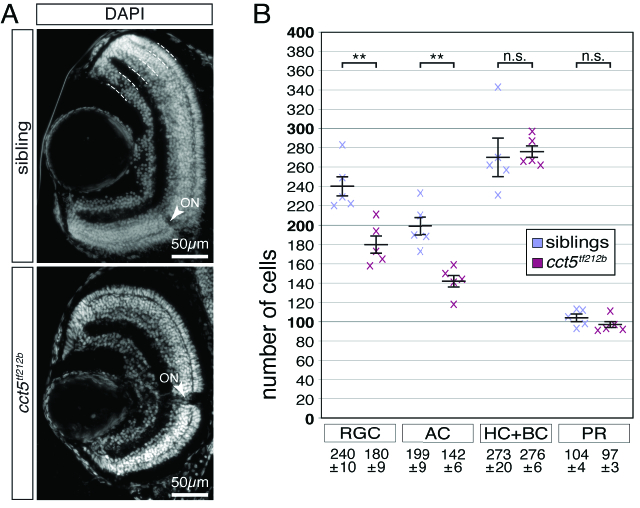
Retinal ganglion and amacrine cells are reduced in number in *cct5 ${}^{tf121b}$* mutants. (A) At six dpf, the retinal nuclei of siblings and *cct5 ${}^{tf121b}$* mutants were marked at the level of the optic nerve using the nuclear stain DAPI. Indicated by the dotted lines, the outer nuclear layer contains the photoreceptor cells (PR). The inner nuclear layer harbours the apical horizontal cells (HC) and the bipolar cells (BC) as well as the basally located amacrine cells (AC). The most basal layer contains the retinal ganglion cells (RGC). Arrowheads indicate optic nerves (ON). (B) Quantification of the cellularity of the different retinal layers within siblings and *cct5 ${}^{tf121b}$* homozygotes at six dpf. The cell number of the RGC and the AC were significantly reduced in *cct5 ${}^{tf121b}$* homozygotes. Per retinal cross section, siblings had 240 ± 10 RGC and 199 ± 9 AC in contrast to 180 ± 9 RGC and 142 ± 6 AC in *cct5 ${}^{tf121b}$* homozygotes. The number of HC and BC as well as PR remained unchanged. Siblings had 273 ± 20 HC and BC as well as 104 ± 24 PR and *cct5 ${}^{tf121b}$* homozygotes had 276 ± 6 HC and BC as well as 97 ± 3 PR. Data are mean ± SEM; **P < 0.01 by Student's *t*-test; n.s., not significant; n, 5.

##  RESULTS

To document the retinal degeneration at larval stages within zebrafish, *cct4 ${}^{-14}$* mutants were utilized that feature loss of TRiC function due to their lack of the Cct4 subunit.^[7]^ Semi-thin cross sections of retinas from *cct4 ${}^{-14}$* homozygotes were generated and stained with Toluidine Blue at six dpf [Figure 1A]. In contrast to the highly organized layers of the sibling retina, within *cct4 ${}^{-14}$* homozygous only a rudimentary lens and dispersed pigments mainly accumulated in a band shape were present. Defined cell structures were absent between a rudimentary lens and clustered pigment, indicating a full degeneration of the *cct4 ${}^{-14}$* retina. In contrast, the retina of *cct5 ${}^{tf212b}$* homozygotes, in which folding of actin but not tubulin is impaired, showed defined nuclei and largely intact layering comparable to their siblings [Figure 1B]. However, whereas signs of retinal degeneration were not obvious on Toluidine Blue-stained sections, the *cct5 ${}^{tf212b}$* homozygous retina seemed reduced in size compared to their sibling retina, indicating that minor defects could be apparent within the *cct5 ${}^{tf212b}$* retina.

To study the retina of *cct5 ${}^{tf212b}$* homozygotes in more detail, the retinal area was quantified on H&E-stained cross sections. Quantification of the retinal area revealed that the retina of *cct5 ${}^{tf212b}$* homozygotes was significantly reduced in size compared to their siblings at three dpf as well as six dpf [Figure 2A]. To further study the reduced retinal size of *cct5 ${}^{tf212b}$* homozygotes, cells in the S phase were labeled using a single 1-hr pulse of the thymidine analogue bromodeoxyuridine (BrdU) prior to fixation at three dpf. Subsequent analysis of BrdU-labeled cells on cross sections revealed that although the location of the proliferating cells within the retinal ciliary marginal zone was not altered in *cct5 ${}^{tf212b}$* homozygotes [Figure 2B], significantly more cells were proliferating during the 1-hr BrdU pulse within *cct5 ${}^{tf212b}$* homozygotes compared to siblings [Figure 2C]. To assess if the significantly smaller *cct5 ${}^{tf212b}$* retinal area despite their higher proliferation rate could be attributed to increased cell death, apoptotic cells were labeled on sections using the TUNEL assay (TdT-mediated dUTP-biotin nick end labeling). With an average of 1.2 ± 0.3 TUNEL-positive cells per retinal cross section, apoptotic cells were rarely found on retinal sections of three-dpf-old siblings [Figure 2D]. In contrast, sections from *cct5 ${}^{tf212b}$* homozygotes harbored significantly more TUNEL-positive cells with an average of 60 ± 6 per section, indicating that apoptosis was greatly enhanced in *cct5 ${}^{tf212b}$* mutants (*n* = 10, *P* < 0.0001). In summary, the significantly reduced size of the retinal area of *cct5 ${}^{tf212b}$* homozygotes could be attributed to the greatly enhanced apoptosis that could not be compensated by the significantly higher proliferation within the ciliary marginal zone.

Residual actin-based thin filaments are formed within the sarcomeres of *cct5 ${}^{tf212b}$* skeletal muscle cells, as TRiC only enhances actin folding but is not absolutely required.^[7]^ In order to unveil F-actin filament assembly within the *cct5 ${}^{tf212b}$* retina, cross sections were stained with the F-actin marker phalloidin and counterstained with DAPI at three dpf. As F-actin mainly locates on the inner surface of cell membranes, the inner and the outer plexiform layers of the sibling retina are strongly marked by phalloidin due to the high neurite densities within these regions [Figure 3A]. In comparison, the phalloidin signal of both plexiform layers appeared weaker in *cct5 ${}^{tf212b}$* homozygotes [Figure 3A]. To quantify the signal intensity of phalloidin from both genotypes, staining of retinal sections and imaging were performed under constant conditions for both *cct5 ${}^{tf212b}$* homozygotes and siblings. Subsequent quantification of the brightness of the IPL revealed that the mean signal intensity from *cct5 ${}^{tf212b}$* homozygotes was significantly reduced compared to their siblings, indicating a reduced level of F-actin filaments within *cct5 ${}^{tf212b}$* mutants. Taken together, in accordance with the reduced actin folding detected in skeletal muscle of *cct5 ${}^{tf212b}$* homozygotes,^[7]^ biogenesis of F-actin filaments was also impaired within the retina, although F-actin filaments were still assembled within *cct5 ${}^{tf212b}$* mutants.

Using the nuclear stain DAPI, the laminar organization of the retina can be visualized that includes the outer nuclear layer, the inner nuclear layer, and the ganglion cell layer [Figure 4A]. The inner nuclear layer can further be subdivided into the basal positioned amacrine cells and the apical compartment with the bipolar and horizontal cells.^[12]^ To further characterize the retinal defects, the cell number within individual retinal layers were counted at six dpf. Quantification of the cellularity of the outer nuclear layer with photoreceptor cells as well as the apical inner nuclear layer with the horizontal cells and the bipolar cells revealed that the number of these cell types remained unchanged between siblings and *cct5 ${}^{tf212b}$* mutants [Figure 4B]. However, quantification of the cellularity of the basal inner nuclear layer and the ganglion cell layer revealed that both layers of *cct5 ${}^{tf212b}$* homozygotes contained significantly less nuclei compared to their siblings, suggesting that amacrine cells as well as retinal ganglion cells were diminished within *cct5 ${}^{tf212b}$* homozygotes [Figure 4B]. Taken together, these results indicate that specifically the amacrine and the retinal ganglion cells of *cct5 ${}^{tf212b}$* mutants are affected by the reduced biogenesis of F-actin filaments.

##  DISCUSSION

Analysis of zebrafish *cct5 ${}^{tf212b}$* mutants revealed that their retinas were reduced in size and their level of F-actin filaments. Enhanced proliferation was not able to compensate for the enhanced apoptosis and numbers of the amacrine and the retinal ganglion cells were depleted.

LCA is a severe congenital retinal dystrophy with over 38 disease-causing variants, including *CCT2*.^[1,2]^ Mutations in the zebrafish ortholog Cct2 as well as other TRiC subunits have been reported to result in retinal degeneration.^[7,8][13]^ Retinal ganglion and amacrine cells are reduced in number in *cct5 ${}^{tf121b}$* mutants. (A) At six dpf, the retinal nuclei of siblings and *cct5 ${}^{tf121b}$* mutants were marked at the level of the optic nerve using the nuclear stain DAPI. Indicated by the dotted lines, the outer nuclear layer contains the photoreceptor cells (PR). The inner nuclear layer harbours the apical horizontal cells (HC) and the bipolar cells (BC) as well as the basally located amacrine cells (AC). The most basal layer contains the retinal ganglion cells (RGC). Arrowheads indicate optic nerves (ON). (B) Quantification of the cellularity of the different retinal layers within siblings and *cct5 ${}^{tf121b}$* homozygotes at six dpf. The cell number of the RGC and the AC were significantly reduced in *cct5 ${}^{tf121b}$* homozygotes. Per retinal cross section, siblings had 240 ± 10 RGC and 199 ± 9 AC in contrast to 180 ± 9 RGC and 142 ± 6 AC in *cct5 ${}^{tf121b}$* homozygotes. The number of HC and BC as well as PR remained unchanged. Siblings had 273 ± 20 HC and BC as well as 104 ± 24 PR and *cct5 ${}^{tf121b}$* homozygotes had 276 ± 6 HC and BC as well as 97 ± 3 PR. Data are mean ± SEM; **P < 0.01 by Student's *t*-test; n.s., not significant; n, 5. However, these mutants were full or partial loss-of-function mutants of individual TRiC subunits resulting in full or partial loss of TRiC function. Consequently, these studied mutants featured deficiencies in folding of both actin and tubulin, the main substrates of TRiC. Accordingly, retinal degeneration within *cct4*-deficient mutants, with only a rudimentary lens and clustered pigment remaining, was confirmed at six dpf in this study.

In order to specifically distinguish the effects of impaired actin folding from the other functions of TRiC in the context of LCA, the retina of the mutant *cct5 ${}^{tf212b}$* was assessed. Although the size of the retina was reduced at three and six dpf, the six-dpf-old *cct5 ${}^{tf212b}$* retina was organized in defined layers and appeared largely intact. Although proliferation of the *cct5 ${}^{tf212b}$* retina was enhanced, abundant apoptosis was detected as well, suggesting that the smaller retinal area might be attributed to the greatly increased apoptosis. These findings are in accordance with zebrafish *cct2 ${}^{L394H-7del}$* mutants that showed a higher number of retinal S phase cells.^[13]^

Residual folded actin can be detected in the absence of TRiC, suggesting that TRiC enhances the folding of actin but is not absolutely required.^[7]^ Compared to other retinal layers, the IPL contains a high level of F-actin filaments due to its high membrane density with interlaced dendrites from cells of the inner nuclear layer and the retinal ganglion cells. Accordingly, the number of F-actin filaments within the *cct5 ${}^{tf212b}$* IPL was found to be reduced, although F-actin filaments were still detected. The intact organization of the *cct5 ${}^{tf212b}$* retinal layers suggested that areas of low actin contents were not notably affected in *cct5 ${}^{tf212b}$* mutants. Thus, the reduced biogenesis of F-actin filaments predominantly within the IPL could be explained by the high levels of F-actin filaments within this layer that require TRiC to enhance the folding of actin.

Retinal organization was further analyzed by the quantification of the cellularity of individual retinal layers, which revealed that specifically the amacrine cells and the ganglion cells were reduced in numbers. Amacrine cells are interneurons that modulate the signal from the rod photoreceptors to the retinal ganglion cells, which transport the visual information through the optic nerve to the brain. Thus, although ocular function was not further elucidated given the severely impaired musculature of *cct5 ${}^{tf212b}$* that would hamper analysis of behavioral responses to visual stimuli,^[7]^ an impaired functionality of the *cct5 ${}^{tf212b}$* eye resulting in visual impairment would be expected from the disrupted anatomical structure of the *cct5 ${}^{tf212b}$* retina. It is noteworthy that LCA is mainly recognized as a disease of the retinal pigment epithelium and photoreceptors^[14]^ and only two sibling patients with variants in *CCT2* resulting in disruption of TRiC were implicated in LCA to date.^[2]^ Variants in *CCT5* have not been associated with patients suffering from LCA as yet, making further studies necessary to fully understand the consequences of impaired F-actin filament formation for LCA.

Taken together, the analysis of the *cct5 ${}^{tf212b}$* retina demonstrated that defects in the enhancement of actin folding by TRiC could potentially contribute to TRiC-associated LCA and that zebrafish *cct5 ${}^{tf212b}$* mutants might be a valuable tool to study the role of F-actin filaments in the context of LCA.

##  Ethical Considerations

The Monash Animal Service approved the treatment of zebrafish males with N-ethyl-N-nitrosourea (MAS/2009/05) and the maintenance of zebrafish lines (ERM/22161).

##  Financial Support and Sponsorship 

JB was supported by the Angior Family Foundation. The Australian Regenerative Medicine Institute is supported by grants from the State Government of Victoria and the Australian Government.

##  Conflicts of Interest

None declared.
